# Seasonal Variation, Chemical Composition, and PMF-Derived Sources Identification of Traffic-Related PM_1_, PM_2.5_, and PM_2.5–10_ in the Air Quality Management Region of Žilina, Slovakia

**DOI:** 10.3390/ijerph181910191

**Published:** 2021-09-28

**Authors:** Dusan Jandacka, Daniela Durcanska

**Affiliations:** Department of Highway and Environmental Engineering, Faculty of Civil Engineering, University of Zilina, Univerzitná 8215/1, 010 26 Žilina, Slovakia; daniela.durcanska@uniza.sk

**Keywords:** particulate matter, PMF, chemical composition, seasonal variation, non-exhaust emissions

## Abstract

Particulate matter (PM) air pollution in the urban environment is mainly related to the presence of potential sources throughout the year. Road transport is one of the most important sources of PM in the urban environment, because it directly affects pedestrians. PM measurements were performed in the city of Žilina, Slovakia, at various road-traffic-related measurement stations over the course of several years. This paper evaluates changes in the concentration of the fine fraction (PM_2.5_), the ultrafine fraction (PM_1_), and the coarse fraction (PM_2.5–10_) over time. PM concentrations were measured by reference gravimetric method. Significant changes in PM concentrations over time due to the diversification of pollution sources and other, secondary factors can be observed from the analysis of the measured data. PM samples were subjected to chemical analysis inductively coupled plasma mass spectrometry (ICP-MS) to determine the concentrations of elements (Mg, Al, Ca, Cr, Cu, Fe, Cd, Sb, Ba, Pb, Ni, and Zn). The seasonal variation of elements was evaluated, and the sources of PM_2.5_, PM_1_, and PM_2.5–10_ were estimated using principal component analysis (PCA) and positive matrix factorization (PMF). PM_2.5_ (maximum concentration of 148.95 µg/m^3^ over 24 h) and PM_1_ (maximum concentration of 110.51 µg/m^3^ over 24 h) showed the highest concentrations during the heating season, together with the elements Cd, Pb, and Zn, which showed a significant presence in these fractions. On the other hand, PM_2.5–10_ (maximum concentration of 38.17 µg/m^3^ over 24 h) was significantly related to the elements Cu, Sb, Ba, Ca, Cr, Fe, Mg, and Al. High correlation coefficients (r ≥ 0.8) were found for the elements Mg, Ca, Fe, Al, Cd, Pb, and Zn in the PM_1_ fraction, Cd, Pb, and Zn in PM_2.5_, and Ba, Sb, Fe, Cu, Cr, Mg, Al, and Ca in PM_2.5–10_. Using PMF analysis, three major sources of PM (abrasion from tires and brakes, road dust resuspension/winter salting, and combustion processes) were identified for the PM_2.5_ and PM_1_ fractions, as well as for the coarse PM_2.5–10_ fraction. This study reveals the importance of non-exhaust PM emissions in the urban environment.

## 1. Introduction

PM is a common proxy indicator of air pollution. The main components of PM are sulfates, nitrates, ammonia, sodium chloride, black carbon, mineral dust, and water. It consists of a complex mixture of solid and liquid particles of organic and inorganic substances suspended in air. While particles with a diameter of 10 micrometers or less (≤PM_10_) can penetrate and settle deep inside the lungs, particles with a diameter of 2.5 micrometers or less (≤PM_2.5_) are even more harmful, as they can penetrate the pulmonary barrier and enter the bloodstream [1].

Epidemiological studies have shown that exposure to PM emissions—in particular PM_2.5_—is associated with various short-term and long-term adverse health effects, such as increased risks of cardiovascular, respiratory, and developmental conditions, as well as an increased risk of overall mortality [2,3,4]. Oxidative stress caused by metals and organic compounds found in PM emissions is considered to be the main biological mechanism responsible for these adverse health effects. Recent research also suggests that air pollution exacerbates epidemics of coronaviruses such as SARS and COVID-19. Such impacts are greatest in urban areas, where emission levels are highest [5,6,7,8,9,10,11,12].

The concentrations, composition, and nature of air pollutants depend mainly on the period and place that are monitored and evaluated for air quality management purposes. In terms of temporality, the dynamics of such processes can be divided into three basic levels: long-term trends, annual course (seasonal changes), and daily course. In terms of the nature of the locality wherein the air quality is monitored, pollutants are commonly divided into urban, rural, and background pollutants.

The annual course (seasonal changes) usually expresses the interaction between specific human activities (use of a certain type of resource) and meteorological conditions that accompany or determine the nature of pollution (temperature, wind, sunshine, etc.). A typical example is the increase in SO_2_, NO_x_, and aerosol concentrations in winter, when increased concentrations of pollutants are caused not only by higher emissions (more sources in winter), but also by low temperatures and inversions, which facilitate the formation, persistence, and slow deposition of pollutants (meteorological parameters). Seasonal changes in PM concentrations may indicate the presence of different sources of air pollution in a particular area [13,14,15,16,17,18,19,20,21].

The main sources of PM air pollution in urban areas include road transport and domestic heating with wood and coal [14,15,22,23,24,25,26,27,28,29,30]. Emissions of PM from motor vehicles originate from two main sources: the combustion of fossil fuels—whose emissions are released via tailpipe exhaust [25,28,31,32]—and non-exhaust processes, including the degradation of vehicle parts and road surfaces [25,33,34,35,36,37,38,39], as well as the resuspension of road dust. The airborne particulate emissions generated by these processes are defined as non-exhaust PM emissions [25,28,34,38,40,41]. PM emissions from road transport directly affect pedestrians in urban areas, with their concentrations often exceeding safe limits for human health [42,43]. The weight, number, size, and chemistry of the particles affect the toxicity of the PM [44,45,46,47,48,49,50,51].

Non-exhaust emissions of PM are beginning to play a major role in the environment, as exhaust emissions of PM have been successfully reduced by introducing direct measures on vehicles (Table 1) [40].

Regarding the chemical composition of PM [2,18,25,45,49,54,55], evidence is still accumulating on the adverse effects of oxidative stress—which is often associated with transition metals and redox-active organic substances such as quinone—on human health [56,57,58,59]. Vehicle-derived particles have a high oxidation potential [59], and a clear increase in the oxidation potential of PM on the road has been found to be associated with metals from non-exhaust emissions [25,32,40,60] (Table 1). Increases in the oxidative potential of such particles are significant, and the metal components identified as determinants of this oxidative activity are toxic to humans [61]. These results are important because they highlight the contribution of currently unregulated pollutants from non-exhaust emissions to negative health outcomes.

The presence of aluminum and silicon in PM has been associated with health problems—especially respiratory problems [62,63,64]. Other elements—including iron, copper, zinc, and sulfur—have also been shown to be associated with adverse health effects, such as cardiopulmonary oxidative stress, heart rate variability, and tissue damage in vivo [65,66,67]. For example, tests on laboratory animals have shown that combined exposure to tire dust, zinc, and copper in high concentrations can lead to oxidative stress in the heart [68].

The present study focuses on the analysis of data on air pollution by PM in the city of Žilina, Slovakia. PM is the most critical pollutant in the city of Žilina, and it is desirable to study its origin as well as any changes in its composition. The aim of this study was to reveal seasonal changes in PM concentrations and to establish the source structure of this pollutant. 

The city of Žilina has long been included on the list of air quality management areas in Slovakia. Air quality management areas are already recognized as an area with poor air quality identified by using monitoring stations at which measures to improve air quality need to be prioritized. This is mainly based on the exceeding of the safe limits of concentrations of PM_10_ and PM_2.5_ for the protection of public health [69]. Therefore, these measurements were focused on the sampling of three fractions of PM (PM_10_, PM_2.5_, and PM_1_). The measurements were performed at seven different traffic-related urban measuring stations during different years and seasons. The measured values provide us with a picture of changes in PM concentrations over the course of the year, during which various sources of pollution are present in the air quality management area (the city of Žilina). The retrospective identification of sources of PM—based on knowledge of their chemical composition, and the possible origin of individual elements—can be an effective tool in the process of eliminating air pollution in cities. PM was chemically analyzed using ICP-MS to determine the presence of selected metals (Mg, Al, Ca, Cr, Cu, Fe, Cd, Sb, Ba, Pb, Ni, and Zn). These elements can come from a variety of sources. Using multidimensional statistical methods and knowledge of the origins of these elements, the sources of PM were estimated. Some of the methods that can be used for this process include principal component analysis (PCA), factor analysis (FA) [70,71,72,73,74,75,76], and positive matrix factorization (PMF) [76,77,78,79,80]. In some previous studies, we focused on identifying PM_10_ sources using PCA and FA [55,81]. In this study, we focused on the PM_1_, PM_2.5_, and PM_2.5–10_ fractions. The PCA and PMF methods were selected for this study. Using the PCA method, the minimum number of hidden variables is determined along with the maximum number of original data; thus, the dimensionality of the solution is reduced. A number of relevant factors for use in PMF were identified using PCA. Using PMF, elements were divided into factors (sources of PM emissions), and their scores were determined, which were then regressed against concentrations in order to estimate the relative contribution of each source. This study focuses on elucidating the composition of PM sources at traffic-related measuring stations, focusing on the negative potential of the formation of non-exhaust PM emissions, and their overall contribution to PM.

## 2. Materials and Methods

### 2.1. Study Area

**Figure 1:**
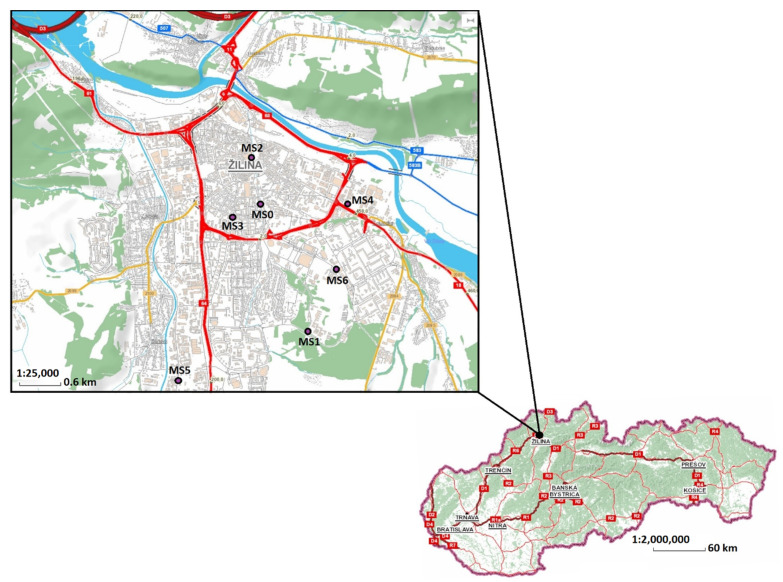
Location of measurement stations in Žilina, Slovakia (Source: https://ismcs.cdb.sk/portal/mapviewer/, accessed on 26 July 2021).

Particulate measurements were performed in the air quality management region in the city of Žilina, Slovakia (Figure 1). Measurements were performed from 2010 to 2019 at different measuring stations—especially near roads—during different measuring periods (Table 2).

A total of 99 PM samples each were taken from the PM_10_, PM_2.5_, and PM_1_ fractions using a nitrocellulose filter, following the gravimetric method. The particulate samples were then subjected to chemical analysis.

### 2.2. Measurements of PM

To establish the amount of ambient PM present in the air, a reference method (the gravimetric method) was applied, pursuant to the STN EN 12341 standard (2016). The sampling was performed using three low-volume flow samplers (LECKEL LVS3 Low-Volume Samplers, Sven Leckel Ingenieurbüro GmbH). Three fractions of PM were monitored concurrently—specifically, PM_10_, PM_2.5_, and PM_1_. The PM was collected on the nitrocellulose filters for 24 h (10 a.m.–10 a.m. the next day). Finally, using this method, we obtained seven 24 h concentrations for each PM fraction at each measurement station (99 filters for each PM fraction—in sum, 297 exposed filters). The nitrocellulose filters had a diameter of 47 mm, and the PM was collected at a fixed airflow rate of 2.3 m^3^/h, after which the mass of PM collected on the filters was determined by means of microbalances, calculated on the basis of the known volume of air obtained during sampling (µg/m^3^). The PM_2.5–10_ concentration was determined as the difference between the fractions PM_10_ and PM_2.5_. All the contamination filters (samples of PM) were used for elemental chemical analysis.

QA/QC: All necessary steps were taken to ensure the quality of the samples and the PM concentrations, including checkweighing. We calibrated all of the measuring instruments as prescribed by the STN EN 12341 standard, and quantified the total uncertainty of the method for PM_1_, PM_2.5_, and PM_10_, consisting of partial uncertainties—sampling of PM by a flow pump and weighing of clean and exposed nitrocellulose filters. This method is maintained as an accredited method at our workplace.

### 2.3. Chemical Analysis of PM

The collected PM contained various elements and compounds. In the next phase of the research, chemical analyses of PM_10_, PM_2.5_, and PM_1_ were performed. Particulate matter was captured on cellulose nitrate (CN) membrane filters. We focused on monitoring 12 elements, which we selected based on their likely origin (source) (Mg, Al, Ca, Cr, Cu, Fe, Cd, Sb, Ba, Pb, Ni, and Zn). Each of these elements comes from a specific source [22,24,25,28,34,54,55,82]. The concentrations of the elements were determined in each sample of PM_10_, PM_2.5_, and PM_1_. The concentrations of elements in the PM_2.5–10_ were determined as the difference between the concentrations of the element in the PM_10_ and PM_2.5_ fractions.

To identify and determine the concentrations of elements in the PM samples, spectroscopic methods were utilized (inductively coupled plasma mass spectrometry (ICP-MS)). The analyses of the 297 filters (99 filters for each PM fraction) and the determination of elements present in the PM_10_, PM_2.5_, and PM_1_ fractions were performed in accordance with the STN EN 14902 standard (2006).

The filters were digested in Teflon vessels with nitric acid (HNO_3_) at a temperature of 230 °C, using a high-temperature and high-pressure SW-4 microwave digestion system (Berghof, Germany). Samples were transferred and appropriately diluted before element determinations on a triple-quadrupole ICPMS (Agilent 8800 Triple-Quadrupole ICP-MS, Japan). The tests were carried out in the laboratory of the Transport Research Centre in Brno, Czech Republic. The final concentration of each element (ng/m^3^) was determined as the difference between the detected concentration of the element in the sample (decomposed exposed filter) and the concentration of the element in the blank (decomposed clean filter).

QA/QC: Chemical analyses were performed using the accredited ICP-MS method within the Transport Research Center in Brno, Czech Republic. Appropriate certified reference materials (CRMs) as calibration standards were used to determine the concentrations of these chemical elements. Measurement uncertainties were determined for individual elements, and were subsequently used in PMF analysis as inputs to the calculations. The QA/QC of this method is an exclusive part of the institution’s accredited laboratory.

### 2.4. Data Analysis

The concentrations of PM_10_, PM_2.5–10_, PM_2.5_, and PM_1_, along with the various tested elements, were subjected to various mathematical analyses. Data matrices measuring 13 variables × 99 measurements were constructed, which contained the concentrations of both the elements and the respective PM fraction.

An important factor in air quality monitoring is the time variation of pollutants. For the purposes of this study, we observed the time variation of PM and the elements that it contains. For this purpose, we used the computer programming language *R* with the open-air air pollution analysis package [83,84].

*R* is a computer programming language developed specifically for the purposes of analyzing data (R-project). It is variously described as a statistical system, a system for statistical computation and graphics, and an environment for data analysis and statistics.

**Figure 2:**
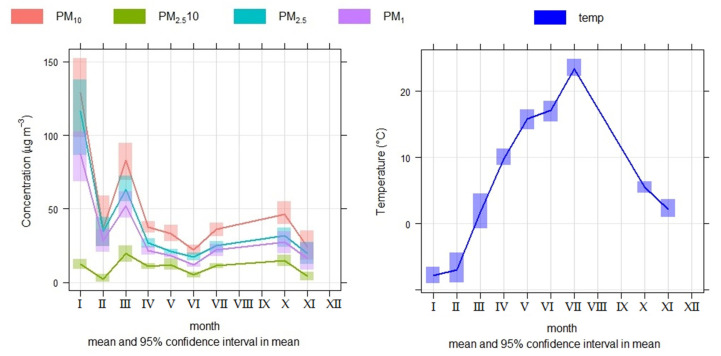
Seasonal variation in PM evaluated from measurements in the city of Žilina.

**Figure 3:**
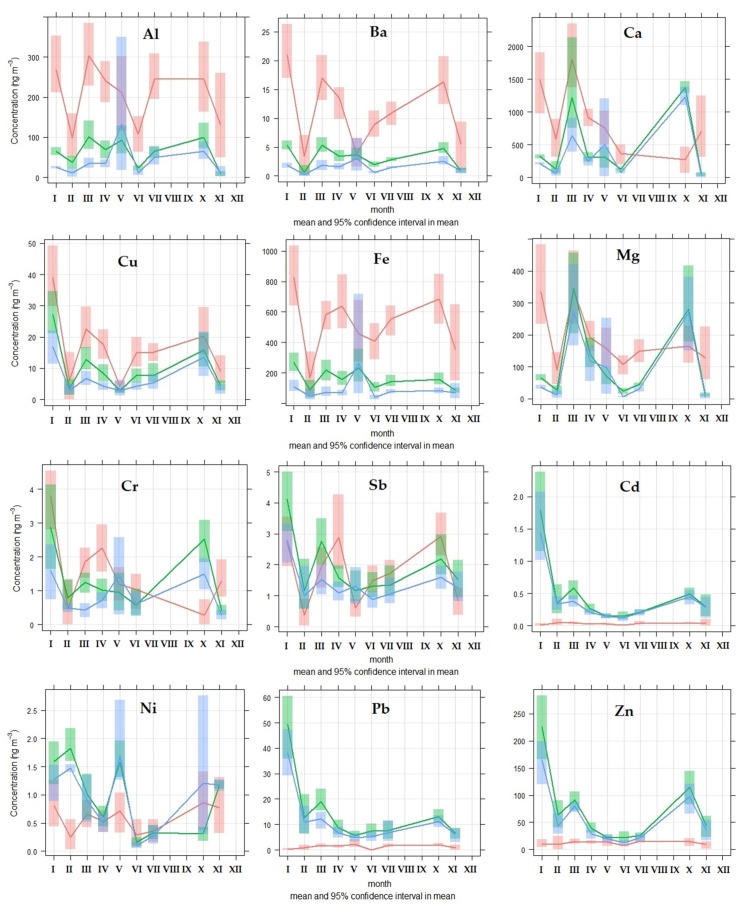
Seasonal variation of concentrations of elements in PM fractions, evaluated based on measurements taken in the city of Žilina (Note—PM_2.5–10_: red; PM_2.5_: green; PM_1_: blue).

The *timeVariation* function produces four plots: day of the week variation, mean hour of the day variation, a combined hour of the day/day of the week plot, and a monthly plot. We mainly used a monthly plot in order to determine the annual course of concentrations of PM and its constituent elements (Figure 2 and Figure 3). The plots also show the 95% confidence interval of the mean. These uncertainty limits can be helpful when trying to determine whether one candidate source is different from another. The uncertainty intervals were calculated through bootstrap re-sampling, which provides better estimates than the application of assumptions based on normality—especially when there are few data available. The function can consider one or more input variables. It should also be noted that variables other than pollutant concentrations can be considered, e.g., meteorological data. The *timeVariation* function is probably one of the most useful functions for the analysis of air pollution.

**Figure 4:**
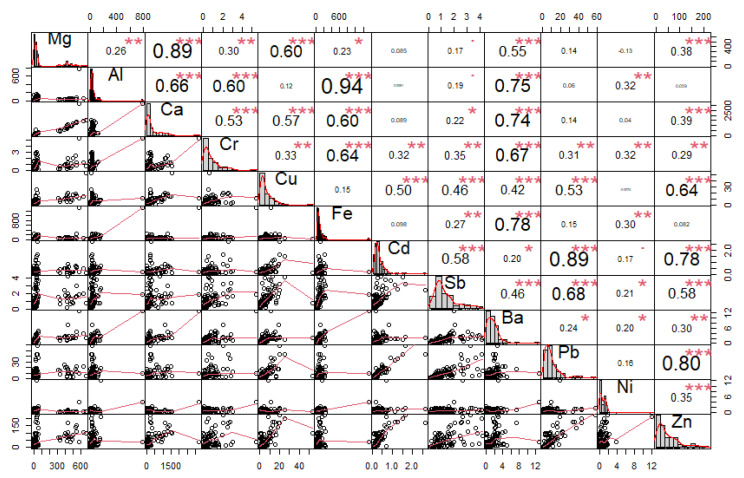
Correlation matrix of elements found in the PM_1_ fraction from measurements taken in the city of Žilina (*p*-values (0, 0.001, 0.01, 0.05, 0.1, 1) <=> symbols (“***”, “**”, “*”, “.”, “ “)).

**Figure 5:**
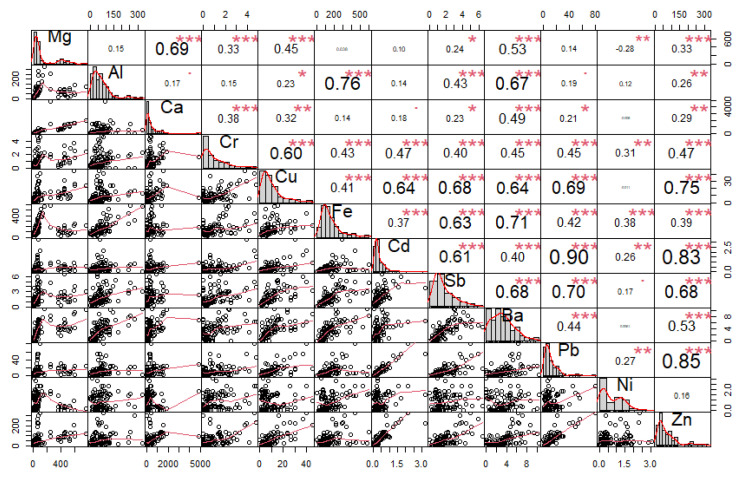
Correlation matrix of elements found in the PM_2.5_ fraction from measurements taken in the city of Žilina (*p*-values (0, 0.001, 0.01, 0.05, 0.1, 1) <=> symbols (“***”, “**”, “*”, “.”, “ “)).

**Figure 6:**
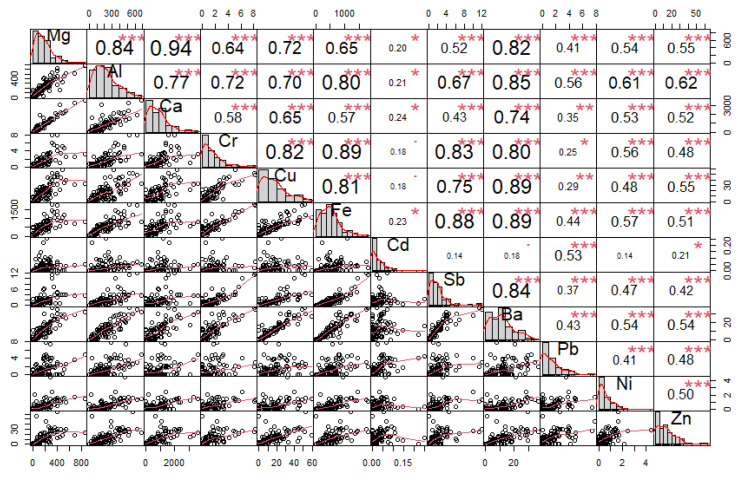
Correlation matrix of elements found in the PM_2.5–10_ fraction from measurements taken in the city of Žilina (*p*-values (0, 0.001, 0.01, 0.05, 0.1, 1) <=> symbols (“***”, “**”, “*”, “.”, “ “)).

Furthermore, correlation analysis was used, where the relationship between individual elements was identified (Figure 4, Figure 5 and Figure 6).

We also used Pearson’s correlation (*r*), which measures a linear dependence between two variables (*x* and *y*). This is also known as a parametric correlation test, because it depends on the distribution of the data. It can be used only when *x* and *y* are normally distributed. The plot of *y* = *f*(*x*) is known as the linear regression curve.

(1) $$r=\frac{\sum \left(x-m_{x}\right)\left(y-m_{y}\right)}{\sqrt{\sum {\left(x-m_{x}\right)}^{2}\sum {\left(y-m_{y}\right)}^{2}}}$$

where:

- *x* and *y* are two vectors of length *n*;
- *m_x_* and *m_y_* correspond to the means of *x* and *y*, respectively.

The *p*-value (significance level) of the correlation can be determined by using the correlation coefficient table for the degrees of freedom: *d_f_ = n* − 2, where *n* is the number of observations in the *x* and *y* variables, or by calculating the *t* value as follows:

(2) $$t=\frac{r}{\sqrt{1-r^{2}}}\sqrt{n-2}$$

where the corresponding *p* value is determined using the *t* distribution table for *d_f_ = n* − 2. If the *p* value is <0.05, then the correlation between *x* and *y* is significant.

The correlation coefficient ranges between −1 and 1:

−1 indicates a strong negative correlation: this means that every time *x* increases, *y* decreases;
- 0 means that there is no association between the two variables (*x* and *y*);
- 1 indicates a strong positive correlation: this means that *y* increases with *x*;
- The function *chart.Correlation()* in the package *PerformanceAnalytics* in *R* can be used to display a chart of a correlation matrix;
- The output of the correlation analysis performed in *R* is shown in Figure 4, Figure 5 and Figure 6, which contain the following information:
- The distribution of each variable is shown on the diagonal;
- At the bottom of the diagonal, the bivariate scatter plots are displayed with a fitted line;
- At the top of the diagonal, the value of the correlation plus the significance level are shown as stars;
- Each significance level is associated with a symbol: *p*-values (0, 0.001, 0.01, 0.05, 0.1, 1) <=> symbols (“***”, “**”, “*”, “.”, “ “)

In the next phase of data analysis, two methods of multidimensional statistical analysis were used—PCA and PMF [55,76,77,78,85]. These methods lead to a reduction in the size of the original task, i.e., transformation of the original variables (elements) into a smaller number of latent variables, so as to preserve as much information as possible about the original data. The results illustrate the interconnection of some elements in the data matrix, along with the subsequent interpretation of their structure based on individual factors—namely, the source of the PM.

**Figure 7:**
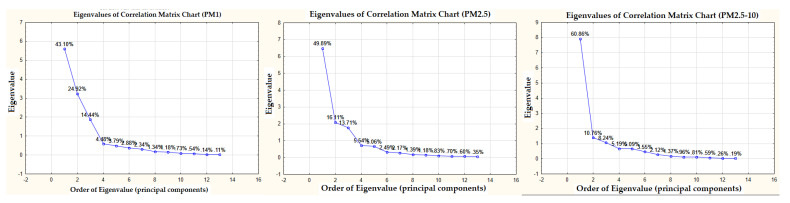
Graphs of ‘‘foothills”—eigenvalues based on PCA.

The primary goal of PCA is the transformation of the original characters of *x_j_*, *j* = 1, …, *m* into a smaller number of latent variables *y_j_*. These latent variables possess more appropriate and comprehensive properties: their presence is less significant, they capture and represent almost the entire variability of the original characteristics, and their properties are mutually uncorrelated. Latent variables are regarded as the main components, and they represent linear combinations of former variables. The first principal component (PC) *y*_1_ describes the greatest part of the variability and, hence, the distribution of the original data, the second principal component *y*_2_, on the other hand, describes the second-greatest part of the variability, capturing the distribution not accounted for within *y*_1_, etc. (Figure 7). Mathematically speaking, the first PC is viewed as a linear combination of input characters that covers the greatest variability, spread between the other linear combinations. In the matrix form, we establish the PCA model of the following string [55,70,72,75,78,85,86]:

(3) $$X=TP^{T}+E$$

where:

*X*: source matrix;
*T*: matrix of the component score;
*P^T^*: transposed matrix of the component loadings; and
*E:* matrix of residues,

and for the first PC, the following relation prevails:

(4) $$y_{1}=\overset{m}{\underset{j=1}{\sum }}v_{1j}x_{j}$$

where:

*x_j_*: former character, input variable, *j* = 1, …, *m*;
*v*_1*j*_: coefficients of eigenvectors.

Receptor models are mathematical approaches to quantifying the contribution of sources to samples based on the composition or fingerprints of the sources. The composition or speciation is determined using analytical methods appropriate for the medium, and key species or combinations of species are needed to separate impacts. A speciated dataset can be viewed as a data matrix *X* of *i* by *j* dimensions, in which *i* samples and *j* chemical species are measured, with *u* uncertainties. The goal of receptor models is to solve the chemical mass balance (CMB) between measured species concentrations and source profiles, as shown in Equation (5), with the number of factors *p*, the species profile *f* of each source, and the amount of mass *g* contributed by each factor to each individual sample [23,55,70,71,72,74,85,86]:

(5) $$x_{ij}=\overset{p}{\underset{k=1}{\sum }}g_{ik}f_{kj}+e_{ij}$$

where *e_ij_* is the residual for each sample/species.

To establish the appropriateness of the factor analysis adopted here, the KMO (interval 0–1) and MSA (interval 0–1) criteria were calculated. In accordance with these criteria, the adoption of factor analysis was substantiated.

The CMB equation can be solved using multiple models, including EPA CMB, EPA Unmix, and EPA Positive Matrix Factorization (PMF). PMF is a multivariate factor analysis tool that decomposes a matrix of speciated sample data into two matrices: factor contributions (*G*), and factor profiles (*F*). These factor profiles need to be interpreted by the user in order to identify the source types that may be contributing to the sample, using measured source profile information and emission or discharge inventories. The method is briefly reviewed here, and described in greater detail elsewhere [76,77,78,79,80]. Results are obtained under the constraint that no sample can have significantly negative source contributions. PMF uses both sample concentration and user-provided uncertainty associated with the sample data to weigh individual data points. This feature allows analysts to account for the confidence in the measurement. For example, data below detection limits can be retained for use in the model, with the associated uncertainty adjusted so that these data points have less influence on the solution than measurements above the detection limit. Factor contributions and profiles are derived by the PMF model minimizing the objective function *Q*:

(6) $$Q=\overset{n}{\underset{i=1}{\sum }}\overset{m}{\underset{j=1}{\sum }}{\left[\frac{x_{ij}-\sum_{k=1}^{p}g_{ik}\times f_{kj}}{u_{ij}}\right]}^{2}$$

## 3. Results

### 3.1. Time Variation Analysis

The analysis of time variation was used to investigate seasonal changes in PM concentrations—especially the PM_2.5_, PM_1_, and PM_2.5–10_ fractions. The analysis showed that the concentrations of PM_2.5_ and PM_1_ tend to be significantly higher in periods with low temperatures (Figure 2). The significance of the effect of temperature on PM_2.5_ and PM_1_ concentrations was subjected to correlation analysis. The correlation coefficients produced the following values: for PM_2.5_ vs. temperature, r = −0.54; and for PM_1_ vs. temperature, r = −0.52. Correlations were significant at the level of *p* < 0.05. The high concentrations in the cold season were mainly due to the presence of seasonal sources, such as local heating, along with suitable conditions for the formation of secondary particles in the air [87]. On the other hand, the PM_2.5–10_ fraction shows an even distribution throughout the year (Figure 2). Road transport contributes greatly to this fraction—especially non-exhaust PM emissions and road dust resuspension [22,25,27,28,38,40,41,45,54,88,89,90,91]; the value found in this study was a contribution of 76.7%. This PM fraction is affected evenly throughout the year by road traffic volume, as well as meteorological factors such as wind speed and rainfall [92]. These factors generate changes in PM concentrations via dry or wet deposition.

The statistical evaluation of all measured concentrations of PM during the mentioned periods is shown in Table 3. The maximum concentration of PM_2.5_ was measured on 29 January 2012 (148.95 µg/m^3^), at which time the concentration of PM_1_ was 110.51 µg/m^3^.

The concentrations of elements in PM are also subject to seasonal changes. We evaluated changes in the concentrations of elements in PM_2.5_, PM_1_, and PM_2.5–10_. The intention was to distinguish between groups of elements that may predominate in the PM_2.5_ and PM_2.5–10_, and vice versa. Elements that predominate in the PM_2.5–10_ (Al, Ba, Ca, Cu, Fe, and Mg) can be formed by mechanical wear from permanent sources over the course of the year, such as from road transport, e.g., non-exhaust emissions and road dust (year-round sources) [25,28,34,40]. On the other hand, there are also elements that predominate in the PM_2.5_ (Cd, Ni, Pb, and Zn), whose concentrations increase significantly (>100%) during the heating season (Table 4). Here, the influence of a seasonal source of PM is assumed—local heating [26,31,73,76,82]. If we use the criterion of the beginning of the heating season in Slovakia, when the average daily temperature is <13 °C (Act on Thermal Energy No. 657/2004 Coll. and Decree of the Ministry of Economy of the Slovak Republic No. 152/2005 Coll., on the specified time and on the specified quality heat supply for the final consumer, respectively), we found an increase in the average concentrations of PM_2.5_ and PM_1_ of 81.4% and 76.2%, respectively, compared to the average concentrations during the non-heating season (average daily temperature > 13 °C). On the other hand, PM_2.5–10_ does not show such a change (Figure 2).

The average concentrations of Mg, Ca, and Cd increased during the heating season in PM_2.5–10_, while Mg, Ca, Cr, Cu, Cd, Sb, and Pb concentrations increased in PM_2.5_ and PM_1_ (Table 4; Figure 3). The greatest change can be observed for Mg and Ca in the PM_2.5_ fraction (Table 4). The average concentrations of Mg (174.94 ng/m^3^) and Ca (555.14 ng/m^3^) in the PM_2.5_ fraction were calculated by comparing the values obtained at temperatures < 13 °C to the average concentrations of Mg (57.45 ng/m^3^) and Ca (249.83 ng/m^3^) obtained at temperatures > 13 °C. This phenomenon is related to winter road maintenance, which ensures the mitigation of defects in the passibility and stairness of roads caused by winter weather conditions. Compounds such as sodium chloride, calcium chloride, magnesium chloride, and others are used as defrosting agents. The purpose of the use of magnesium chloride-based spreading materials on roads is to eliminate faults in the passibility of roads, as a step in their winter maintenance, so as to help road managers to maintain the roads’ normal condition. This helps to increase safety on roads in winter, as well as assisting with troubleshooting of roadworthiness, and providing an outlet for the ecological disposal of magnesium chloride. However, elements contained in spreading materials enter the air, water, and soil, affecting their natural state [93].

An increase in concentration during the heating season is also observed for the elements Cd, Pb, and Zn—mainly in the PM_2.5_ fraction; this change is probably related to local heating and combustion processes [30,76,78]. On the other hand, we can also observe a decrease in the concentrations of some chemical elements during the heating season, which is reflected mainly in the PM_2.5–10_ fraction for the elements Al, Cr, Cu, Fe, Sb, Ba, Pb, Ni, and Zn (Table 4, Figure 3). Some of these elements represented in the PM_2.5–10_ fraction are related to road transport—specifically, to non-exhaust emissions of PM [22,25,34,39,40]. The primary source of PM in the city of Žilina is road transport during the non-heating season—mainly non-exhaust PM emissions (e.g., road surface abrasion, tires, brakes, and road dust resuspension), which, under the more suitable dispersion conditions during the non-heating season, can enter the ambient air more easily and can stay there for some time. This claim is also supported by subsequent analyses of PCA and PMF.

### 3.2. Elemental Correlation Analysis

The performed correlation analysis of the concentrations of elements in individual fractions of PM revealed some internal bonds between the variables. The elements Mg/Ca, Fe/Al, and Cd/Pb/Zn in the PM_1_ fraction show high correlation coefficients (r ≥ 0.8) (Figure 4). In the PM_2.5_ fraction, the elements Cd/Pb/Zn show high correlation coefficients (Figure 5). Significant correlations between Ba, Sb, Fe, Cu, Cr, Mg, Al, and Ca were found in the PM_2.5–10_ (Figure 6). Non-exhaust emissions of PM (e.g., road surface abrasion, tire abrasion, brake and clutch wear) were primarily responsible for the formation of the PM_2.5–10_ [25,28,34,39]. This is further supported by significant correlations between elements, which may indicate a common source of PM [25,28,31,34,40].

### 3.3. PCA and PMF Analyses

Furthermore, PMF (EPA PMF 5.0 program [77]) was used to explain the internal bonds between elements in PM. An uncertainty dataset that assigns an uncertainty value to each species was also used. Uncertainties determined for individual elements using the ICP-MS method, along with uncertainties determined for PM using the gravimetric method, were used for the calculation. The result of the analysis was the identification of various sources of PM_1_, PM_2.5_, and PM_2.5__–__10_.

Using PCA, three main components were extracted for the PM_1_, PM_2.5_, and PM_2.5–10_ fractions, each of which reached an eigenvalue > 1. In the case of PM_1_, these three principal components characterized 82.5% of the variance of the original data; in the case of PM_2.5_, they characterized 79.7%; and in the case of PM_2.5–10_, they characterized 79.9% (Figure 7). Subsequently, these three principal components were used in the PMF analysis for all of the PM fractions.

The bootstrap (BS), displacement (DISP), and BS-DISP error estimation were used for every PM model:

1. DISP results show that the solution is stable because no swaps are present;
2. BS results—mapping over 80% of the factors indicates that the BS uncertainties can be interpreted and the number of factors may be appropriate;
3. BS-DISP results—the number of swaps is one for two factors, which indicates some ambiguity between the factors. The number of swaps is low.

On the basis of the results of the criteria, factor analysis can be applied for PM_1_ (KMO = 0.7, reflecting average correlation between the variables; MSA > 0.5, indicating that the *i*-th variable is at least moderately predicted by the others), for PM_2.5_ (KMO = 0.8, reflecting good correlation between the variables; MSA > 0.6, indicating that the *i*-th variable is at least moderately predicted by the others) and for PM_2.5–10_ (KMO = 0.8, reflecting good correlation between the variables; MSA > 0.6, indicating that the *i*-th variable is at least moderately predicted by the others).

**Figure 8:**
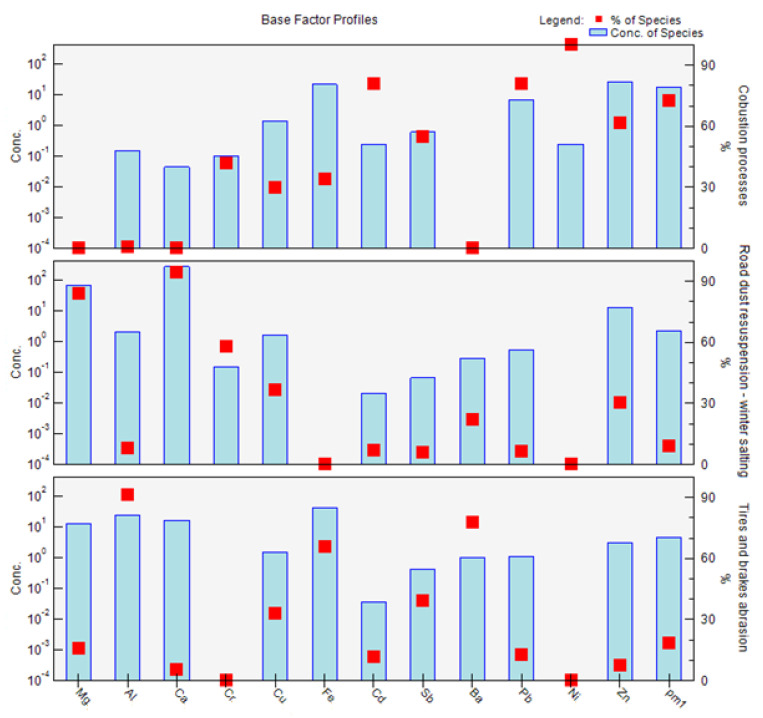
Factor profiles: division of elements into individual factors, and their characterization, from measurements of PM_1_ taken in the city of Žilina.

The factor composition obtained via PMF analysis for the PM_1_ fraction is shown in Figure 8. The profile graph for the PM_1_ fraction (Figure 8) shows the concentration of each species (element) assigned to the factor as a pale blue bar, and the percentage of each species assigned to the factor as a red box. The concentration line corresponds to the left *y*-axis, which is a logarithmic scale; the percentage of species corresponds to the right *y*-axis. Factor 1 is therefore characterized mainly by the elements Cd, Pb, Ni, and Zn if we take into account the classification criterion > 60%. In the case of factor 2, the major elements are Mg, Ca, and Cr. Factor 3 is characterized by the elements Al, Fe, and Ba. The factors are characterized according to the representation of individual elements in their profiles according to the possible source of the combination of elements, the time distribution of PM and elemental concentrations over the course of the year, and the interrelationships between the elements (correlations) (Figure 8). 

**Figure 9:**
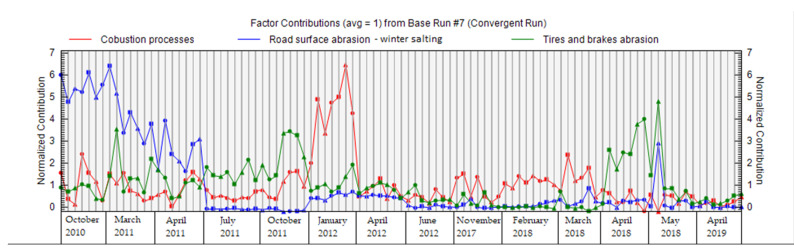
Contribution of factors during individual periods of PM_1_ measurements in the city of Žilina.

Figure 9 shows the contribution of all factors to the total weight of the sample; this graph can be used to verify the names of factors, and to determine the distribution of factors for individual species (elements). The largest contribution of factor 1 was combustion processes in January 2012, during the heating season. This period was characterized by very low temperatures and considerable inversion, indicative of a significant heating season. The main contribution of factor 2—road dust resuspension and winter salting—to PM_1_ concentrations was most pronounced in the periods of October 2010, March 2011, and April 2011, when spreading materials were widely used to ensure road safety; these periods were characterized by low temperatures and frequent icing on the roads. The main contribution of factor 3—abrasion of tires and brakes—does not show such extremes as factors 1 and 2, and is mostly uniform across different measurement periods (Figure 9).

Factors 1–3 contribute to the PM_1_ variable as follows—factor 1: 72.4%; factor 2: 9.1%; factor 3: 18.4%—according to the PMF results. It is clear that combustion processes make a significant contribution to the formation of PM_1_. The contributions of individual factors are characteristic of the periods and places where they are most pronounced.

**Figure 10:**
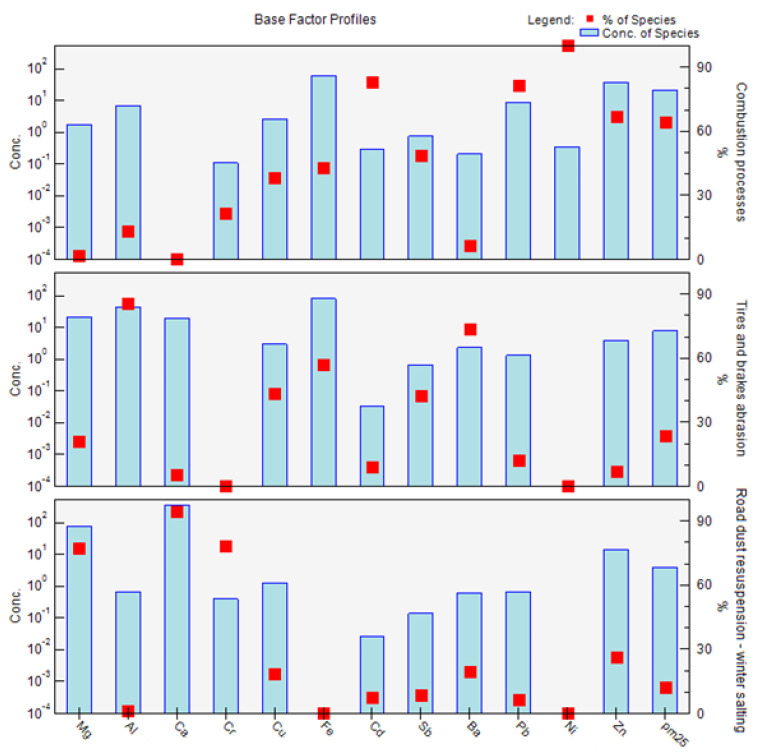
Factor profiles: division of elements into individual factors, and their characterization, from measurements of PM_2.5_ taken in the city of Žilina.

**Figure 11:**
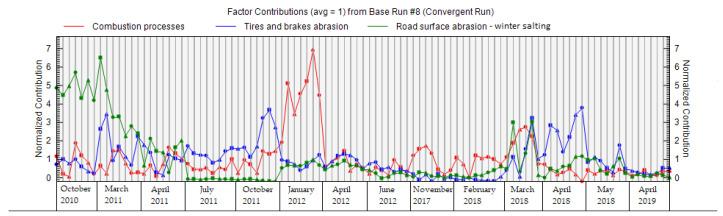
Contribution of factors during individual periods of PM_2.5_ measurements in the city of Žilina.

The factors obtained by PMF analysis for the PM_2.5_ fraction are shown in Figure 10. Factor 1 is mainly characterized by the elements Cd, Pb, Ni, and Zn if we take into account the sorting criterion > 60%. In the case of factor 2, the major elements are Al, Fe, and Ba. Factor 3 is characterized by the elements Mg, Ca, and Cr. The factors have a different order for PM_2.5_ than for PM_1_. The order of factors is according to their significance for different PM fractions (Figure 10). The contributions of factors to the PM_2.5_ fraction at different measurement periods are similar to those of the PM_1_ fraction (Figure 11). Due to the representation of these elements in individual factors, and the time variation of the contributions of these factors, the sources of PM_2.5_ are characterized as follows: factor 1: combustion processes; factor 2: abrasion of tires and brakes; factor 3: road dust resuspension and winter salting (Figure 10). Factors 1–3 contribute to the PM_2.5_ as follows—factor 1: 64.4%; factor 2: 23.6%; factor 3: 12.1%—according to the PMF results. It is clear that combustion processes also make a sizeable contribution to the formation of PM_2.5_, with a slight increase in the significance of non-exhaust emissions in this PM fraction. A study by Jandacka and Durcanska (2019 [81]) revealed a factor with a combination of elements K, S, Cd, Pb, Zn for PM_10_ using FA, and this fact was also taken into account when naming the factor 1.

**Figure 12:**
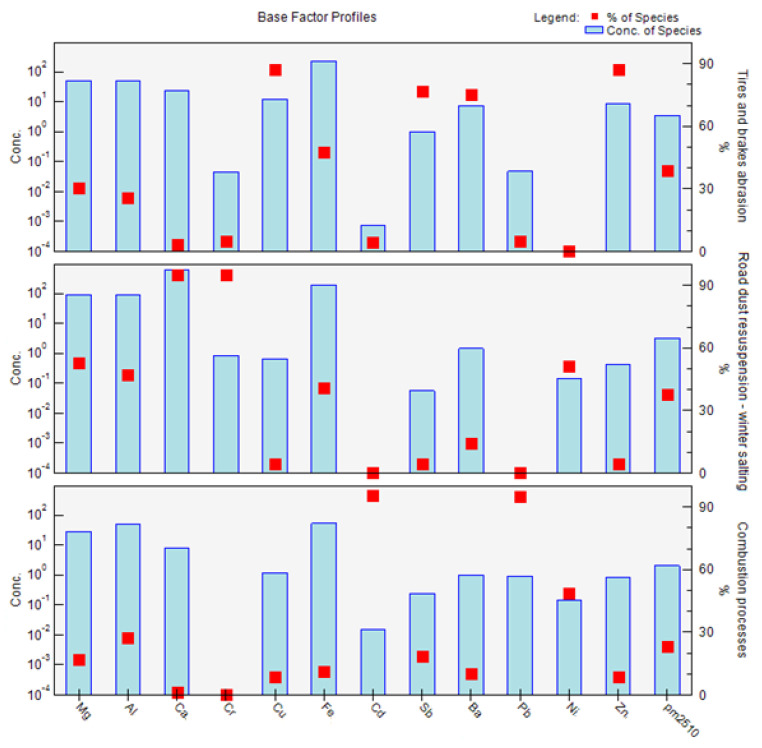
Factor profiles: division of elements into individual factors, and their characterization, from measurements of PM_2.5–10_ taken in the city of Žilina.

**Figure 13:**
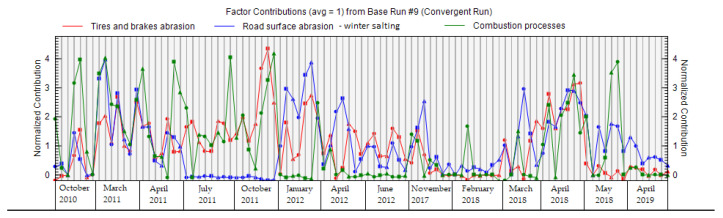
Contribution of factors during individual periods of PM_2.5–10_ measurements in the city of Žilina.

The factors obtained via PMF analysis for the PM_2.5–10_ fraction are shown in Figure 12. Factor 1 is mainly characterized by the elements Cu, Sb, Ba, and Zn if we take into account the sorting criterion > 60%. In the case of factor 2, the major elements are Mg, Ca, and Cr. Factor 3 is characterized by the elements Cd and Pb. The results of the analysis point to the fact that PM_2.5_ and PM_2.5–10_ have different properties, show different seasonal changes and, most importantly, that there is a different share of resources in their production. In the case of the PM_2.5–10_ fraction, the main role is played by the sources from which PM is released in a rather mechanical way, i.e., non-exhaust PM emissions. The PMF analysis revealed a higher significance of the non-exhaust sources in PM_2.5–10_: factor 1—abrasion of tires and brakes;—and factor 2—road dust resuspension and winter salting—has the greatest impact in this PM fraction. Factor 3—combustion processes—also shows a partial effect on this PM fraction (Figure 12). Differences between the PM_2.5_ and PM_2.5–10_ fractions can also be observed in the case of seasonal factor contributions, where we did not observe such significant extremes in the case of PM_2.5–10_ as in the cases of PM_2.5_ and PM_1_ (Figure 13). Factors 1–3 contribute to the variable PM_2.5–10_ as follows—factor 1: 39.0%; factor 2: 37.7%; factor 3: 23.3%—according to the PMF results. The Mg and Ca elements are concentrated in a factor 2, these elements being bound to the PM_2.5–10_. They can deposit on the surface of the road from salts of winter spreading material, soil dust [94]. Subsequently, they are released into the air by resuspension.

## 4. Conclusions

Due to the diversity of particulate sources, their properties can be different, and their impact on the environment and human health is determined by these properties. The task of research in this area is primarily to clarify conditions of air quality, and changes therein. Increased particulate matter concentrations are a problem—especially in urban areas, where large numbers of people are concentrated.

The present study is devoted to the issue of PM occurring in the air (namely, its concentration, distribution, seasonal changes, chemical composition, and sources). Analysis of the time variation of PM revealed some changes in concentrations over the course of the year. PM_2.5–10_ has an even distribution throughout the year, which represents the presence of a permanent source—road transport. PM_2.5_ and PM_1_ show higher concentrations—especially during the heating period (significant correlation of concentrations with air temperature)—to which the seasonal source of particulate matter (local heating) contributes. Moreover, at low temperatures, secondary particles are formed from gaseous pollutants, increasing PM concentrations [87]. Similar results regarding higher concentrations during the heating season have been obtained in various studies [13,16,17,95]. High concentrations of PM_2.5_ (maximum concentration of 148.95 µg/m^3^ over 24 h) and PM_1_ (maximum concentration of 110.51 µg/m^3^ over 24 h) during the heating season are the result of the presence of several sources of PM in Žilina, Slovakia. The average concentration of PM_2.5–10_ from all measurements represents 23.3% and PM_2.5_ represent 76.7% of the average concentration of the total fraction PM_10_. Seasonal variation revealed an increase in the concentrations of elements (>100%) during the heating season—especially those in the PM_2.5_ fraction (Mg, Ca, Cd, Pb, and Zn).

The performed correlation analysis of the concentrations of elements in individual fractions of PM revealed some internal bonds between variables. The elements Mg/Ca, Fe/Al, and Cd/Pb/Zn in the PM_1_ fraction showed high correlation coefficients (r ≥ 0.8). In the PM_2.5_ fraction, the elements Cd/Pb/Zn showed high correlation coefficients. Significant correlations between Ba, Sb, Fe, Cu, Cr, Mg, Al, and Ca were revealed in the PM_2.5–10_. Pearson’s correlation coefficients can be used to determine the degree of correlation between elements, and can provide information related to the sources and pathways of elements, as presented in various studies [23,73,74].

The objective of this study—identification of the sources of PM_1_, PM_2.5_, and PM_2.5–10_ using PMF—revealed various sources of these pollutants near the monitoring stations. PMF analysis revealed three factors, and their contribution to the production of PM_1_—factor 1: combustion processes (72.7%); factor 2: road surface abrasion (9.1%); and factor 3: abrasion of tires and brakes (18.4%)—PM_2.5_—factor 1: combustion processes (64.4%); factor 2: abrasion of tires and brakes (23.6%); and factor 3: road surface abrasion (12.1%)—and PM_2.5–10_—factor 1: abrasion of tires and brakes (39%); factor 2: road surface abrasion (37.7%); and factor 3: combustion processes (23.3%). PM source identification studies have been performed by different authors using different methods [25,76,78,85,86,96]. However, the interpretation of PM sources is conditioned by the given measuring point, where different PM sources may meet over time. In the case of this study, we used monitoring sites—mainly on roads—to which the interpretation of factors was partly subordinated.

Road dust resuspension/winter maintenance and tire and brake wear were identified as non-exhaust PM emissions via PMF analysis. These non-exhaust PM emissions make a substantial contribution to the composition of PM_2.5–10_, as revealed by various studies [25,28,39]. These two sources of non-exhaust PM emissions contribute to the formation of 27.5% of PM_1_, 35.7% of PM_2.5_, and 76.7% of the PM_2.5–10_.

While emission standards for particulate matter from motor vehicle exhausts are tightening worldwide, PM emissions from non-exhaust processes are mostly unregulated. As a result, the share of PM emissions from non-exhaust sources has increased in recent years, due to a significant reduction in the amount of PM from exhaust emissions. The importance of non-exhaust emissions is also highlighted by this study, while other institutions and workplaces have reached similar conclusions [40]. In further research into traffic-related PM emissions, it is appropriate to extend the measurements of PM and to extend the list of relevant chemical elements, which may improve the analysis and interpretation of the results.

By combining and using the analyses presented in this work, it is possible to define a methodology for identifying sources of particulate matter in the air, which can be of great practical benefit for use in air quality assessment and the application of targeted measures to reduce air pollution by particulate matter in affected areas [97].

## Figures and Tables

**Table 1:** Chemical composition of non-exhaust PM [40].

Non-Exhaust Emissions Category	Main Components (>1% in Mass) *	Measurement Method
Brake wear	Iron, Copper, Barium, Antimony, Zinc, Aluminum, Chromium, Potassium, Titanium, and Magnesium [52]	Brake dynamometer
Tire wear	Zinc, Silicon, and Sulfur [41]	Road simulator
Road wear	Silicon, Calcium, Potassium, and Iron [34,38,39]	Road simulator
Road dust resuspension	Silicon, Calcium, Aluminum, Iron, Potassium, Magnesium, Titanium, Copper, Zinc, and Barium [23,49,53]	Road dust sampling

* Most common tracers are in bold.

**Table 2:** PM measuring stations in the city of Žilina, Slovakia.

Measuring Station	Location	Measuring Periods	Characteristics of the Measuring Place
**Vojtecha Spanyola Street (MS0)**	49°13′06.8″ N, 18°44′36.2″ E	19–25 October 2010; 8–14 March 2011; 11–17 April 2011; 7–14 July 2011; 13–19 October 2011; 26 January–1 February 2012; 16–22 April 2012; 7–13 June 2012	Placed in the vicinity of Vojtecha Spanyola Street near habitation, shopping centers, and a hospital.
**Univerzitná Street (MS1)**	49°12′6.61″ N, 18°45′14.24″ E	14–20 November 2017	Placed in the vicinity of the crossroads of Univerzitná Street and Veľký Diel Street near the University of Žilina campus.
**A. Hlinka Square (MS2)**	49°13′29.08″ N, 18°44′31.10″ E	22‒28 February 2018	Square, with pedestrian zone, connected to the streets by road traffic.
**Komenského Street (MS3)**	49°12′58.64″ N, 18°44′15.63″ E	1‒7 March 2018	Placed in the vicinity of the crossroads of Komenského Street, Suvorovova Street, and Juraja Fándlyho Street near residential buildings, educational buildings, and a public administration building.
**Košická Street (MS4)**	49°13′8.30″ N, 18°45′36.80″ E	19–25 April 2018	Important city traffic hub and the biggest city crossroads, near the city’s heating plant and shopping centers.
**Štrková Street (MS5)**	49°11′35.27″ N, 18°43′37.12″ E	9–15 May 2018	Placed in the vicinity of Štrková Street. High volume of heavy road traffic, including trucks.
**Vysokoškolákov Street (MS6)**	49°12′38.20″ N, 18°45′29.15″ E	9–15 April 2019	Placed in the vicinity of Vysokoškolákov Street, near habitation, shopping centers, and a hospital.

**Table 3:** Descriptive statistics of PM concentrations from measurements in the city of Žilina.

	Fraction of PM			
	PM_10_	PM_2.5–10_	PM_2.5_	PM_1_
	Values of descriptive statistics (µg/m^3^)			
Min	3.91	0.00	3.78	2.75
Max	158.54	38.17	148.95	110.51
Median	37.30	10.07	28.75	23.99
Mean	47.87	11.16	36.80	29.81
Var	1062.41	64.54	826.04	493.90
Std. dev	32.59	8.03	28.74	22.22
Skewness	1.75	1.05	2.17	1.82
Kurtosis	5.75	4.33	7.67	6.25

**Table 4:** Changes in the concentrations of elements in individual PM fractions during the heating season compared to the non-heating season.

PM Fraction	Percentage Change in the Concentrations of Elements during the Heating Season Compared to the Non-Heating Season (%)											
	Mg	Al	Ca	Cr	Cu	Fe	Cd	Sb	Ba	Pb	Ni	Zn
PM_2.5–10_	+7.3	−14.6	+10.9	−49.0	−8.4	−28.9	+12.1	−43.4	−5.7	−16.0	−8.6	−17.0
PM_2.5_	+204.5	−22.8	+122.2	+22.9	+54.2	−29.9	+192.4	+20.4	−8.1	+127.8	+3.8	+239.2
PM_1_	+246.6	−51.5	+48.9	−27.2	+61.1	−48.6	+152.5	+8.0	−31.0	+113.9	+15.3	+262.0

Note: “+”: increase in concentration during the heating season; “−“: decrease in concentration during the heating season.

## Data Availability

Not applicable.
